# Single-cell transcriptomic analysis reveals cellular and molecular changes in EGFR-positive lung adenocarcinoma before and after Furmonertinib treatment

**DOI:** 10.1007/s13258-025-01706-y

**Published:** 2025-11-27

**Authors:** Jian Chen, Minghui Cai, Lifei Meng, Ze Hong, Wentao Hu

**Affiliations:** 1 Department of Thoracic Surgery, The First Affiliated Hospital of Ningbo University, Ningbo, China; 2 Department of Cardiothoracic Surgery, Taizhou Hospital of Zhejiang Province Affiliated to Wenzhou Medical University, Linhai, China

**Keywords:** Lung adenocarcinoma_1_, EGFR_2_, Furmonertinib_3_, Single-cell RNA-seq_4_, Tumor microenvironment_5_

## Abstract

**Background:**

Lung adenocarcinoma (LUAD) frequently harbors activating mutations in the epidermal growth factor receptor (EGFR), making EGFR tyrosine kinase inhibitors (EGFR-TKIs) a critical component of targeted therapy. Although third-generation EGFR-TKIs, such as Furmonertinib, have improved outcomes for patients with EGFR-mutant LUAD, drug resistance and tumor adaptation remain major challenges. The cellular and molecular mechanisms underlying response and adaptation to Furmonertinib, particularly within the tumor microenvironment (TME), are not fully understood.

**Methods:**

We performed single-cell RNA sequencing on tumor and paired paracancerous tissues from EGFR-positive LUAD patients before and after Furmonertinib treatment, integrating both public and in-house datasets. We systematically analyzed changes in cellular composition, gene expression profiles, pathway enrichment, and ligand–receptor-mediated cell–cell communication.

**Results:**

Furmonertinib treatment led to a marked reduction in tumor cell proportion and profound remodeling of the TME. There was an increase in T cell infiltration, particularly CD4^+^ T cells, and a decrease in exhausted CD8^+^ T cells, indicating a shift toward a less immunosuppressive microenvironment. Cancer-associated fibroblasts and monocytes were also enriched post-treatment. Tumor cells exhibited increased EGFR expression, along with transcriptomic reprogramming characterized by upregulation of signaling, immune, and differentiation pathways, and downregulation of metabolic and protein synthesis genes. Cell–cell communication analysis revealed attenuation of immunosuppressive signaling (such as MIF axis) and enhancement of alternative ligand–receptor interactions, including LAMC1- and EGFR-related pathways.

**Conclusions:**

Our integrative single-cell analysis reveals that Furmonertinib therapy induces significant cellular and molecular changes in EGFR-positive LUAD, including TME remodeling, transcriptomic adaptation, and reprogramming of intercellular communication networks. These findings provide insight into the mechanisms of Furmonertinib response and resistance, and may inform strategies to optimize EGFR-TKI therapy.

**Supplementary Information:**

The online version contains supplementary material available at 10.1007/s13258-025-01706-y.

## Introduction

Lung adenocarcinoma (LUAD) remains the most common histological subtype of non-small cell lung cancer (NSCLC) and is a major cause of cancer-related mortality worldwide (Liu et al. 2023). Activating mutations in the epidermal growth factor receptor (EGFR) gene drive tumorigenesis in a significant subset of LUAD patients, making EGFR tyrosine kinase inhibitors (EGFR-TKIs) a mainstay of targeted therapy (Liu et al. 2024; Zhang et al. 2024). Although first- and second-generation EGFR-TKIs have demonstrated clinical benefit, acquired resistance inevitably emerges, prompting the development and clinical adoption of third-generation EGFR-TKIs, such as Furmonertinib (Darré et al. 2024; Kleczko et al. 2023).

EGFR-TKIs are a class of small-molecule targeted therapies that specifically inhibit the kinase activity of mutant EGFR proteins, thereby blocking aberrant downstream signaling pathways involved in tumor cell proliferation, survival, and metastasis (Tu et al. 2024). First-generation EGFR-TKIs (such as gefitinib and erlotinib) reversibly bind to the ATP-binding site of EGFR and are effective against tumors harboring classical sensitizing mutations (Darré et al. 2024). However, acquired resistance—most commonly driven by the EGFR T790M mutation—inevitably develops during treatment (Darré et al. 2024; Tu et al. 2024). Second-generation TKIs (such as afatinib and dacomitinib) irreversibly inhibit EGFR and other ERBB family members, but offer only modest improvement in overcoming resistance (Darré et al. 2024). Third-generation EGFR-TKIs, including osimertinib and Furmonertinib, are designed to selectively target both activating and T790M resistance mutations, while sparing wild-type EGFR, and have become the standard of care for patients with advanced EGFR-mutant NSCLC (Zhang et al. 2024; Kleczko et al. 2023). Despite these therapeutic advances, resistance to third-generation EGFR-TKIs remains a clinical challenge, highlighting the need for further mechanistic investigation and novel therapeutic strategies.

Recent advances in single-cell RNA sequencing (scRNA-seq) have enabled high-resolution dissection of tumor and microenvironmental heterogeneity, providing new insights into the dynamic cellular and molecular changes induced by targeted therapies (Fan et al. 2022). Several recent studies have leveraged scRNA-seq to demonstrate that EGFR-TKI treatment can profoundly alter the immune landscape and intercellular communication networks within the TME, influencing both anti-tumor immunity and the emergence of drug resistance (Fan et al. 2022; Chen et al. 2024). Recent single-cell RNA-seq studies have shown that EGFR-mutant LUAD possesses a profoundly immunosuppressive tumor microenvironment, characterized by a scarcity of CD8^+^ tissue-resident memory T cells and reduced cell–cell checkpoint signaling, which may contribute to the poor response to immunotherapy (Yang et al. 2022).Additionally, single-cell analyses of early-stage EGFR-mutant LUAD have uncovered pronounced heterogeneity in both tumor and immune cell compartments. The microenvironment is enriched for myeloid cells, exhausted and regulatory T cells, and displays broad immunosuppressive features. Tumor-associated macrophages adopt intermediate polarization states, while intercellular signaling pathways actively promote tumor progression and resistance to therapy (He et al. 2021).

Despite these advances, a comprehensive understanding of how Furmonertinib reshapes the cellular composition, transcriptional states, and cell–cell communication networks in EGFR-positive LUAD remains lacking. In this study, we performed integrative single-cell transcriptomic analysis of tumor and matched paracancerous tissues from EGFR-mutant LUAD patients, including both publicly available and in-house sequenced samples, before and after Furmonertinib treatment. We systematically characterized alterations in cellular composition, gene expression, functional enrichment, and ligand–receptor-mediated cell–cell communication, aiming to reveal the adaptive responses and molecular mechanisms underlying Furmonertinib therapy.

## Material and method

### Patients

A total of twelve samples were included in this study. Five EGFR-positive LUAD tumor samples and five matched paracancerous tissue samples were obtained from the publicly available CRA001963 dataset (https://bigd.big.ac.cn/gsa) (He et al. 2021). In addition, one EGFR-positive LUAD tumor sample and its paired paracancerous tissue were collected from a patient at the First Affiliated Hospital of Ningbo University (Ningbo, China). The patient was first diagnosed with LUAD based on pathological examination, and the presence of an EGFR mutation was confirmed by next-generation sequencing. Following diagnosis, the patient received Furmonertinib treatment. After the patient exhibited a favorable clinical response, both tumor and paracancerous tissues were collected by surgical resection for single-cell RNA sequencing analysis. Written informed consent was obtained, and the study was approved by the Ethics Committee of the First Affiliated Hospital of Ningbo University (Approval No. 2025-Research-018A).

### Preparation of single cell suspension

LUAD tumor tissues and matched paracancerous samples from Furmonertinib-treated patients were collected at the First Affiliated Hospital of Ningbo University. Freshly resected tissues were transferred into MACS Tissue Storage Solution (Miltenyi Biotec, cat. no. 130–100-008) and kept on ice. All samples were processed within two hours after surgical removal. Following brief centrifugation at 50 × g for 1 min at 4 °C, the storage solution was discarded. Tissues were finely minced into ~ 1 mm^3^ fragments using sterile scissors and digested in pre-warmed RPMI-1640 medium containing collagenase IV (1–2 mg/mL) and DNase I (0.1 mg/mL). The digestion was performed in a 37 °C water bath for 30–60 min with gentle agitation, and the suspension was pipetted every 5 min to enhance dissociation. To terminate enzymatic activity, 10% fetal bovine serum (Pricella, cat. no. 164210–50) was added. The cell suspension was filtered through a 40 μm cell strainer (Corning, cat. no. CLS431750) to remove debris and isolate single cells. The filtered cells were centrifuged at 300 × g for 3 min at 4 °C. Red blood cells were lysed using RBC lysis buffer (Roche, cat. no. 11814389 001), followed by another centrifugation at 300 × g for 3 min at 4 °C. The resulting cell pellets were resuspended in PBS containing 0.01% bovine serum albumin (Sigma, cat. no. A1933). Cell viability and concentration were assessed using trypan blue exclusion with a hemocytometer or automated cell counter, and only samples with > 85% viability were used for downstream analyses.

### Single-cell RNA sequencing

Single-cell suspensions with > 90% viability and a concentration of approximately 1,000 cells/μL were loaded into the microfluidic chip of the Chip A Single Cell Kit v2.1 (MobiDrop (Zhejiang) Co., Ltd., cat. no. S050100301) and processed on the MobiNova-100 system (cat. no. A1A40001) to generate gel bead-in-emulsion (GEM) droplets, each encapsulating a single cell and a barcoded gel bead containing millions of oligonucleotides. Light cutting was subsequently performed using the MobiNovaSP-100 system (cat. no. A2A40001) to release the oligos into the reaction mix, enabling mRNA capture and barcoded reverse transcription within droplets. Barcoded cDNAs were then PCR-amplified and used to construct libraries with the High Throughput Single-Cell 3′ Transcriptome Kit v2.1 (cat. no. S050200301) and 3′ Dual Index Kit (cat. no. S050300301). cDNA quality was assessed using the Agilent 2100 Bioanalyzer. The final libraries were sequenced on the MobiNova-100 platform with 150 bp paired-end reads. After quality filtering, raw reads were aligned to the human reference genome (GRCh38), and gene expression matrices were generated using the Mobivision software (v3.2).

### Single-cell RNA analysis

Single-cell RNA-seq data were processed using the R package “Seurat” (version 5.3.0, https://satijalab.org/seurat/) (Hao et al. 2024). Low-quality cells were filtered out by standard quality control procedures. Doublets were identified and removed using DoubletFinder (version 2.0.6). Cells were retained if they had between 200 and 5,000 detected genes (nFeature_RNA), and UMI counts between 500 and 15,000. Cells with mitochondrial gene expression greater than 15% or hemoglobin gene expression greater than 1% were excluded (Fig. S1A). After filtering, gene expression data were normalized, and 2,000 highly variable genes were selected using the variance-stabilizing transformation (VST) method. Principal component analysis (PCA) was performed, and the top 30 principal components were chosen according to the ElbowPlot. After correcting for batch effects, cell clustering was performed using the FindNeighbors and FindClusters functions, and Uniform Manifold Approximation and Projection (UMAP) was used for visualization. Cell types were annotated based on markers from the CellMarker 2.0 (http://117.50.127.228/CellMarker/) database (Hu et al. 2023). In addition, we referred to previously published marker sets reported in Wen et al. 2024 to guide our annotation strategy, ensuring the robustness of cell type identification (Wen et al. 2024). Intercellular communication was analyzed using the CellChat software package by inferring ligand-receptor interactions, evaluating communication probabilities, and identifying key signaling pathways. Differentially expressed genes were identified using the FindMarkers function (padj < 0.05, |log2FC|> 1).

### AUCell

Gene set activity scoring was performed using the R package AUCell (version 1.30.1) (Aibar, 2016). Two gene sets, FIGAROL_EGFR_TKI_DRUG_TOLERANT_CELL_UP and FIGAROL_EGFR_TKI_DRUG_TOLERANT_CELL_DN, representing genes upregulated or downregulated in EGFR-TKI drug-tolerant cells (Figarol et al. 2024), were used as input. The drug-resistant cell signature activity was assessed at the single-cell level using the AUC score.

### Functional enrichment analysis

Differentially expressed genes (DEGs) between tumor cells from the LUAD group and the Furmonertinib-treated group were identified and subjected to Gene Ontology (GO) and Kyoto Encyclopedia of Genes and Genomes (KEGG) pathway enrichment analyses. Enrichment analysis was performed using the R package clusterProfiler (version 4.16.0) (Yu et al. 2012), with org.Hs.eg.db for GO annotation and the KEGG database for pathway annotation. Significantly enriched GO terms and KEGG pathways were defined as those with adjusted *p* value < 0.05. Visualization of enrichment results was conducted using clusterProfiler and ggplot2 (Villanueva et al. 2016).

### Cell–cell communication

Cell–cell communication analysis between the LUAD group and the Furmonertinib-treated group was performed using the R package CellChat (version 1.6.0) (Jin 2022) with the CellChatDB.Human ligand–receptor database. Single-cell RNA-seq data and cell type annotations were used as input. Differential intercellular signaling pathways between the two groups were identified according to the standard workflow. Pathway-level intercellular communication analysis was further conducted using CellChat to identify and compare specific signaling pathways between groups.

### Statistical analysis

In this study, data were standardised using log transformation and batch correction. All analyses and visualisations were conducted with R software (version 5.0.1). For statistical comparisons, Wilcoxon test were used to assess differences between two groups, while one-way ANOVA was applied to evaluate differences among multiple groups. Statistical significance was defined as a p-value of less than 0.05, with significance levels denoted as follows: * (*p* < 0.05), ** (*p* 0.01), and *** (*p* < 0.001).

## Result

### Single-cell transcriptomic profiling of EGFR-positive LUAD before and after Furmonertinib treatment

**Fig. 1:**
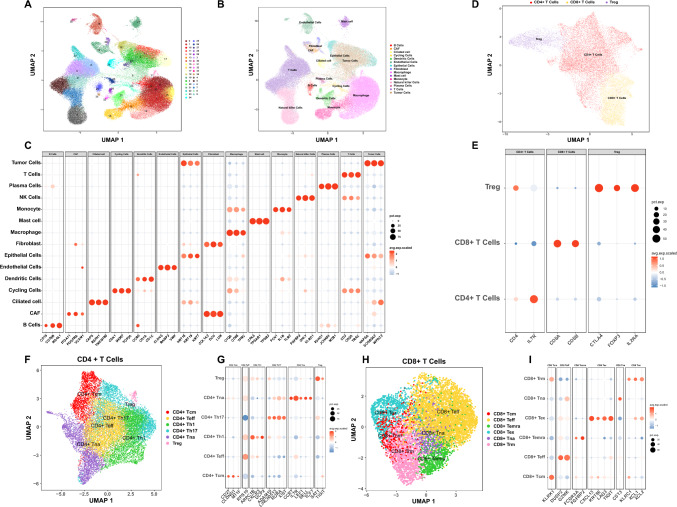
Single-cell transcriptome profiles of EGFR-positive LUAD before and after furmonertinib treatment.** A**,** B** UMAP plots of all cells from 12 samples colored by unsupervised clusters and annotated cell types.** C** Dot plot of canonical marker gene expression used for cell type annotation; dot size represents the percentage of cells expressing the marker, and color indicates average scaled expression.** D** UMAP visualization of T cell populations.** E** Dot plot illustrating expression of canonical marker genes used to identify T cell subsets. Dot size represents the percentage of cells expressing the gene; color indicates average scaled expression.** F** UMAP plot of CD4 + T cells.** G** Dot plot of CD4 + T cells canonical marker gene expression, Dot size indicates percentage of cells expressing the gene, color indicates scaled average expression.** H** UMAP plot of CD8 + T cells.** I** Dot plot of marker gene expression used for CD8 + T cell subset identification

To investigate the cellular landscape and transcriptional heterogeneity of EGFR-positive LUAD in response to Furmonertinib treatment, we performed scRNA-seq on tumor samples and paired paracancerous tissues collected before and after treatment. After quality control and filtering, the ScRNA-seq data of the 12 samples were retained for downstream analysis. Batch effects were corrected using canonical correlation analysis (CCA) (Fig. S1B, C), and all cells were projected into a shared low-dimensional space using UMAP (Fig. 1A). Each cluster was annotated based on the expression of canonical marker genes (CellMarker 2.0), including *KRT18*, *KRT19* and *KRT7* for epithelial cells; *SCGB3A2*, *WFDC2* and *NAPSA* for tumor cells; *CAPS*, *RSPH1* and *TMEM190* for ciliated cells; *CLDN5*, *RAMP2* and *VWF* for endothelial cells; *LUM*, *DCN* and *COL1A2* for fibroblasts; *ITGA11*, *PDGFRA*, and *VCAM1* for cancer-associated fibroblasts (CAFs); *JCHAIN*, *IGHG1*, and *MZB1* for plasma cells; *CD79B*, *MS4A1*, and *CD19* for B cells; *MKI67*, *CDK1* and *TOP2A* for cycling cells; *CD3D*, *TRAC* and *CD2* for T cells; *KLRD1*, *GNLY* and *FGFBP2* for natural killer (NK) cells; *CD68*, *TFRC* and *C1QA* for macrophages; *FCN1*, *IL1B* and *TLR2* for monocytes; *CD1C*, *CD1A* and *CCR6* for dendritic cells; *TPSB2*, *TPSAB1* and *CPA3* for mast cells (Fig. 1B, C). To further explore T cell heterogeneity, T cells were subjected to two rounds of subclustering. In the first round, T cells were classified into CD4^+^ T cells, CD8^+^ T cells, and regulatory T cells (Tregs) (Fig. 1D, E). In the second round, both CD4^+^ and CD8^+^ T cells were further subdivided into distinct functional subsets, including naïve, central memory, effector, and other specialized subpopulations (Fig. 1F, G, H, I).

### Changes in cellular composition following Furmonertinib treatment

**Fig. 2:**
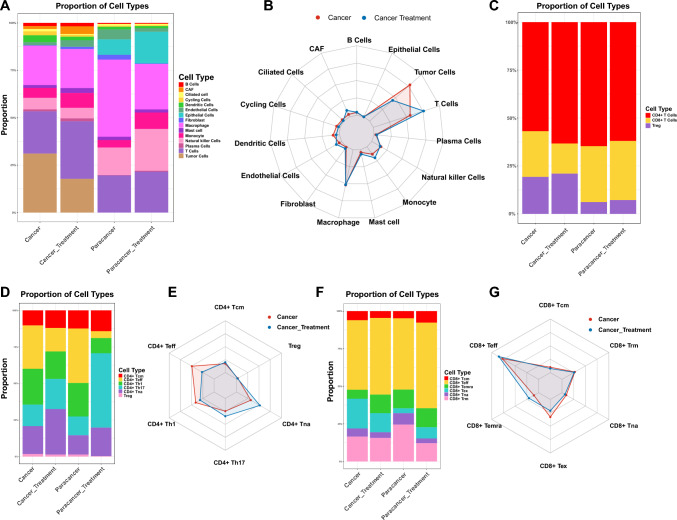
Cell proportion analysis results.** A** Bar plot showing the proportion of each major cell type across four sample groups: cancer, cancer with Furmonertinib treatment, paracancerous, and paracancerous with treatment.** B** Radar plot comparing the average proportions of major cell types between the cancer group and the Furmonertinib treatment group.** C** Bar plot displaying the proportion of CD4 + T cells, CD8 + T cells, and Tregs in cancer, cancer with Furmonertinib treatment, paracancerous, and paracancerous with treatment groups.** D** Bar plot displaying the proportion of each CD4 + T cell subset in different sample groups.** E** Radar plot showing the average proportion of CD4 + T cell subsets between cancer and treatment groups.** F** Bar plot of CD8 + T cell subset proportions in each group.** G** Radar plot of average CD8 + T cell subset proportions in cancer and treatment groups

To investigate the impact of Furmonertinib treatment on the tumor microenvironment, we compared the proportions of major cell types between the cancer group and the treatment group. The results showed that the relative abundance of each cell type changed following therapy. Compared to the cancer group, the proportion of tumor cells exhibited the greatest decrease after Furmonertinib treatment. In contrast, T cells showed the largest increase in proportion. Additionally, the proportions of cancer-associated fibroblasts (CAFs) and monocytes increased in the treatment group, while the proportion of dendritic cells decreased. These findings indicate that Furmonertinib treatment is associated with marked changes in both immune and stromal cell composition in EGFR-positive LUAD (Fig. 2A, B).

To explore the effects of Furmonertinib treatment on the T cell compartment, we analyzed the distribution of T cell subsets in the cancer and treatment groups. The results showed that the proportion of CD4^+^ T cells increased in the treatment group compared to the cancer group, while the proportion of CD8^+^ T cells decreased (Fig. 2C). Within the CD4^+^ T cell population, the proportions of CD4^+^ effector T cells (Teff) and CD4^+^ Th1 cells decreased, whereas the proportions of CD4^+^ Th17 cells and CD4^+^ naïve T cells (Tna) increased after treatment (Fig. 2D, E). For CD8^+^ T cells, the proportion of CD8^+^ exhausted T cells (Tex) decreased in the treatment group, while the proportions of CD8^+^ terminally differentiated effector memory T cells (Temra) and CD8^+^ effector T cells (Teff) increased (Fig. 2F, G).

### EGFR expression differences between the cancer and treatment groups

**Fig. 3:**
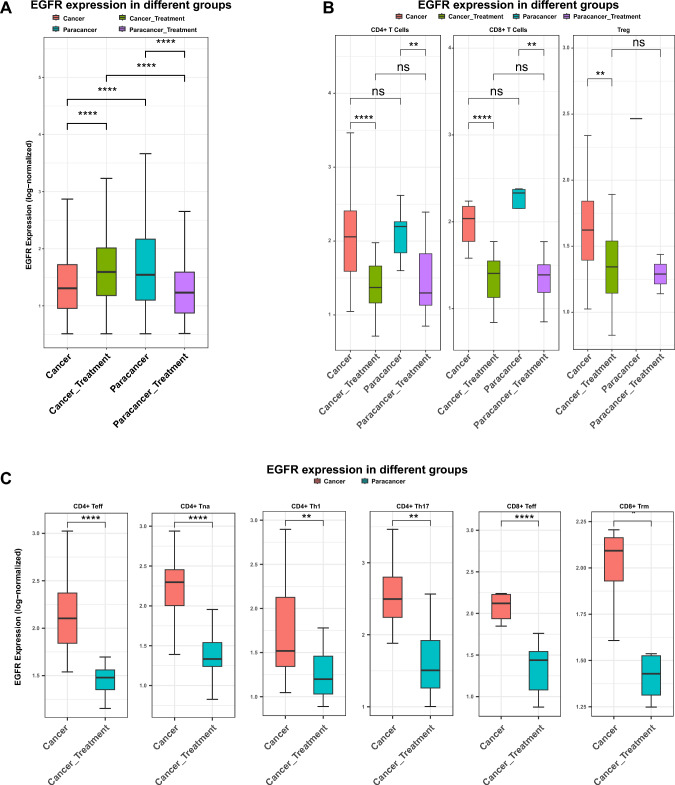
Heterogeneity and EGFR expression of CD4 + and CD8 + T cell subsets in EGFR-positive LUAD.** A** Boxplot illustrating EGFR expression levels (log-normalized) in tumor cells across the four groups. Statistical significance was assessed by Wilcoxon test; *p  < 0.05, **p  < 0.01, ***p  < 0.001, ****p < 0.0001.** B** Boxplots of EGFR expression (log-normalized) in CD4 + T cells, CD8 + T cells, and Tregs across different groups. Statistical comparisons were performed using the Wilcoxon test; ns, not significant; **p < 0.01, ****p < 0.0001.** C** Boxplots showing EGFR expression (log-normalized) in selected CD4 + and CD8 + T cell subsets between cancer and treatment groups. Statistical comparisons were performed using Wilcoxon test; **p < 0.01, ****p < 0.0001

We compared EGFR expression levels between tumor cells and all other cell types in the cancer and treatment groups, and between epithelial cells and all other cell types in the paracancerous and treatment groups. The analysis showed that EGFR expression was significantly higher in the treatment group compared to the corresponding control group (*p* < 0.0001) (Fig. 3A). EGFR expression also exhibited heterogeneous dynamics across different cell populations. In most immune and stromal cell types—including NK cells, T cells (*p* < 0.0001), mast cells (*p* < 0.05), monocytes (*p* < 0.05), dendritic cells (*p* < 0.01), and B cells (*p* < 0.05)—EGFR expression was lower in the treatment group than in the cancer group. In contrast, macrophages (*p* < 0.0001), cycling cells (*p* < 0.01), tumor cells (*p* < 0.0001), and plasma cells (*p *< 0.01) displayed higher EGFR expression in the treatment group (Fig. S2). Further analysis of T cell populations revealed that EGFR expression in CD4^+^ T cells (*p* < 0.0001), CD8^+^ T cells (*p* < 0.0001), and Tregs (*p* < 0.01) was consistently lower in the treatment group compared to the cancer group (Fig. 3B). This trend remained evident within T cell subsets: CD4^+^ Teff (*p* < 0.0001), CD4^+^ naïve T cells (*p* < 0.0001), CD4^+^ Th1 (*p* < 0.01), CD4^+^ Th17 (*p* < 0.001), CD8^+^ Teff (*p* < 0.0001) and CD8^+^ tissue-resident memory T cells (*p* < 0.05) all exhibited decreased EGFR expression after Furmonertinib treatment (Fig. 3C).

### Differential gene expression and pathway enrichment in tumor cells between cancer and treatment groups

**Fig. 4:**
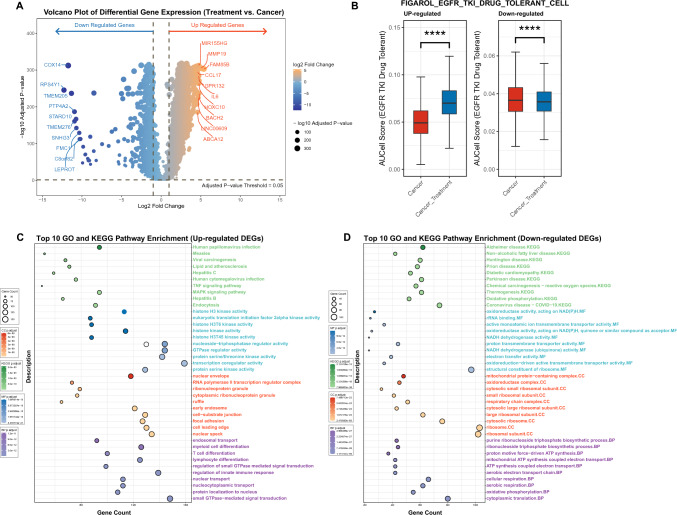
Differential gene expression, drug-resistance signature activity, and functional enrichment in tumor cells following Furmonertinib treatment.** A** Volcano plot of DEGs; blue indicates downregulated genes, orange indicates upregulated genes, and dot size reflects statistical significance.** B** Box plot of the difference in AUC scores of drug-resistant gene sets between the tumor group and the treatment group.** C** Top 10 GO and KEGG enrichment for upregulated DEGs.** D** Top 10 GO and KEGG enrichment for downregulated DEGs, bubble size shows gene count and color indicates adjusted *p* value

Differential gene expression analysis between tumor cells from the cancer group and the treatment group identified 3,016 upregulated genes and 950 downregulated genes following Furmonertinib treatment (Fig. 4A). Functional enrichment analysis showed that upregulated genes were mainly associated with small GTPase-mediated signal transduction, nuclear transport, regulation of innate immune response, lymphocyte and T cell differentiation, endosomal transport, cell signaling, and transcriptional regulation (Fig. 4C). KEGG pathway analysis further indicated enrichment in endocytosis, MAPK signaling pathway, TNF signaling pathway, and various viral infection-related pathways (Fig. 4C). In contrast, downregulated genes were primarily enriched in cytoplasmic translation, oxidative phosphorylation, cellular respiration, ATP synthesis, ribosomal structure, and mitochondrial function (Fig. 4D). These genes were also involved in KEGG pathways such as oxidative phosphorylation, thermogenesis, and several neurodegenerative diseases (Fig. 4D). Overall, these findings indicate that Furmonertinib treatment induces extensive transcriptomic reprogramming in tumor cells. The observed upregulation of genes involved in cell signaling, immune response, and differentiation pathways suggests that tumor cells may activate alternative signaling cascades and immune-related processes in response to targeted therapy. Meanwhile, the downregulation of genes related to mitochondrial respiration, oxidative phosphorylation, and protein synthesis reflects a marked suppression of cellular metabolism and biosynthetic capacity. This coordinated shift in gene expression may represent an adaptive response to EGFR inhibition, aiming to limit cell proliferation and energy production while promoting survival mechanisms.

### AUCell analysis of drug-tolerant gene signatures

Our analysis demonstrated that, compared to the cancer group, the Furmonertinib treatment group exhibited significantly higher AUC scores for the upregulated drug-tolerant gene set and significantly lower AUC scores for the downregulated gene set (Fig. 4B). At the single-cell level, all major cell types in the treatment group showed consistently elevated AUC values for the upregulated gene set relative to the cancer group, indicating a widespread activation of drug-tolerant transcriptional programs following treatment (Fig. S3A). For the downregulated gene set, B cells, cycling cells, monocytes, plasma cells, and tumor cells in the treatment group displayed significantly reduced AUC values, while ciliated cells, macrophages, mast cells, and T cells exhibited increased AUC values after Furmonertinib exposure (Fig. S3B).

### Alterations in cell–cell communication between groups

**Fig. 5:**
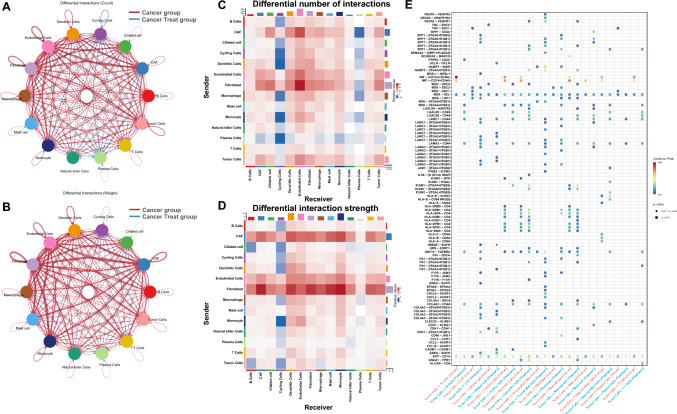
Differential cell–cell communication patterns between the cancer and treatment groups in EGFR-positive LUAD.** A**,** B** Circos plots showing differences in intercellular interaction counts (**A**) and weights (**B**) between the two groups. Thicker and darker lines indicate greater increases in the treatment group, and each node color represents a different cell type. Red lines represent upregulation in the cancer group, and blue lines represent upregulation in the cancer treat group.** C**,** D** Heatmaps displaying changes in the number (**C**) and weight (**D**) of cell–cell interactions. The x-axis indicates receivers, and the y-axis indicates senders.** E** Dot plot showing selected differentially expressed ligand–receptor pairs; dot color indicates interaction strength and size reflects statistical significance. In (**A**–**D**), blue indicating decreases and red indicating increases after treatment

Cell–cell communication analysis using CellChat revealed extensive remodeling of intercellular signaling networks after Furmonertinib treatment (Fig. 5A, B). In the treatment group, the number of incoming interactions to cycling cells and plasma cells increased compared to the cancer group, while the strongest interaction strengths were mainly observed in fibroblasts from the cancer group (Fig. 5C, D). Dot plot analysis of differentially expressed ligand–receptor pairs highlighted specific communication axes altered by therapy. Following Furmonertinib exposure, the MIF signaling axis was weakened in several immune cell types, including T cells, cycling cells, dendritic cells, macrophages, mast cells, and natural killer (NK) cells. In contrast, LAMC1-mediated interactions were enhanced in cancer-associated fibroblasts (CAFs), ciliated cells, endothelial cells, and fibroblasts. Additionally, interactions mediated by AREG–EGFR, EREG–EGFR, and HBEGF–EGFR were increased, particularly involving CAFs and ciliated cells (Fig. 5E).

## Discussion

Our findings indicate that Furmonertinib treatment leads to significant remodeling of the tumor microenvironment in EGFR-positive LUAD. The marked decrease in tumor cell proportion reflects effective tumor suppression by EGFR-TKI therapy. Meanwhile, the increase in T cells, especially CD4^+^ subsets, and the reduction in exhausted CD8^+^ T cells suggest a relief of immunosuppression and a partial restoration of anti-tumor immune responses. These immune shifts are consistent with recent single-cell studies showing that targeted therapies can enhance immune infiltration and reprogram the tumor immune landscape (Fan et al. 2022; Pan et al. 2023). Additionally, the increased proportions of CAFs and monocytes indicate that stromal and myeloid cells are also reshaped by treatment, which may contribute to tissue remodeling or influence future resistance (Zhang et al. 2023a, b).

The observed increase in EGFR expression in tumor cells after Furmonertinib treatment is consistent with known adaptive resistance mechanisms in EGFR-mutant LUAD. Previous studies have shown that EGFR-TKI therapy can select for residual tumor cell subpopulations with higher EGFR expression, which are more likely to survive under drug pressure (Moghal et al. 2023). This selection effect, combined with compensatory feedback signaling that upregulates EGFR transcription, may lead to enrichment of EGFR-high tumor cells following treatment (Zhang et al. 2023a, b). In contrast, the decreased EGFR expression in most immune and stromal cells, including T cells, NK cells, and dendritic cells, suggests a suppression or rebalancing of EGFR signaling in these compartments under TKI therapy (Pan et al. 2023; Wang et al. 2024). The divergent responses among different cell types highlight the heterogeneity of microenvironmental adaptation to targeted therapy.

The observed transcriptomic changes in tumor cells following Furmonertinib treatment are consistent with adaptive reprogramming mechanisms reported in targeted therapy resistance. The upregulation of genes related to alternative signaling pathways, immune responses, and cellular differentiation suggests that tumor cells may attempt to compensate for EGFR inhibition by activating parallel or bypass signaling networks, as well as mounting a stress or immune response (Moghal et al. 2023). which shows that drug-tolerant cancer cells often rely on alternative growth and survival pathways to persist under continuous therapeutic pressure (Yan et al. 2023). Conversely, the downregulation of genes involved in oxidative phosphorylation, mitochondrial function, and protein synthesis reflects a metabolic shift toward reduced energy consumption and biosynthetic activity ( Moghal et al. 2023; Bao et al. 2022). Such metabolic suppression is characteristic of persister cells and may represent a survival strategy, allowing tumor cells to evade apoptosis and maintain viability during drug exposure.

Cell–cell communication analysis revealed that Furmonertinib treatment resulted in extensive remodeling of intercellular signaling networks within the tumor microenvironment. The increased number of incoming interactions to cycling and plasma cells suggests that these populations may become more responsive or functionally active following therapy, possibly contributing to tissue remodeling or adaptive responses (Fan et al. 2022). The observation that fibroblasts displayed the strongest interaction strengths in the untreated cancer group is consistent with the established role of CAFs as central hubs in the TME, supporting tumor growth and immune evasion (Zhang et al. 2023a, b; Moghal et al. 2023). Importantly, Furmonertinib exposure attenuated MIF-mediated signaling among multiple immune cell types, which may facilitate the relief of immunosuppression and enhance anti-tumor immunity (Fan et al. 2022). In contrast, the enhancement of LAMC1-mediated signaling in CAFs, ciliated cells, and endothelial cells indicates increased matrix remodeling and tissue adaptation (Zhang et al. 2023a, b). Furthermore, the upregulation of AREG–EGFR, EREG–EGFR, and HBEGF–EGFR interactions, especially involving CAFs and ciliated cells, highlights the activation of alternative EGFR-related paracrine pathways, which are implicated in resistance, repair, and tumor cell survival during EGFR-TKI therapy (Moghal et al. 2023).

## Conclusion

This single-cell analysis reveals that Furmonertinib treatment in EGFR-positive LUAD leads to a marked reduction in tumor cells and significant remodeling of the tumor microenvironment, including increased T cell infiltration and changes in stromal and myeloid cell populations. Tumor cells show adaptive transcriptomic reprogramming, with increased EGFR expression and activation of alternative signaling and immune pathways, while downregulating metabolic and protein synthesis genes. Cell–cell communication networks are also extensively reshaped, with reduced immunosuppressive signaling and enhanced alternative ligand–receptor interactions. These results provide new insights into the mechanisms of Furmonertinib response and resistance, and may inform strategies to optimize EGFR-TKI therapy.

## Supplementary Information

Below is the link to the electronic supplementary material.

Supplementary file1 (PDF 4348 KB)

Supplementary file2 (PDF 535 KB)

Supplementary file3 (PDF 206 KB)

## Data Availability

Due to privacy associated with human-derived samples, the single-cell RNA sequencing data generated in this study are not publicly available. Data access may be granted upon reasonable request to the corresponding author.
