# Supplementary figures and images for: Single-cell transcriptomic analysis reveals cellular and molecular changes in EGFR-positive lung adenocarcinoma before and after Furmonertinib treatment
Source: Genes Genomics. 2025 Nov 27;48(3):327–37. doi: 10.1007/s13258-025-01706-y (PMC12932405; doi:10.1007/s13258-025-01706-y)

**A**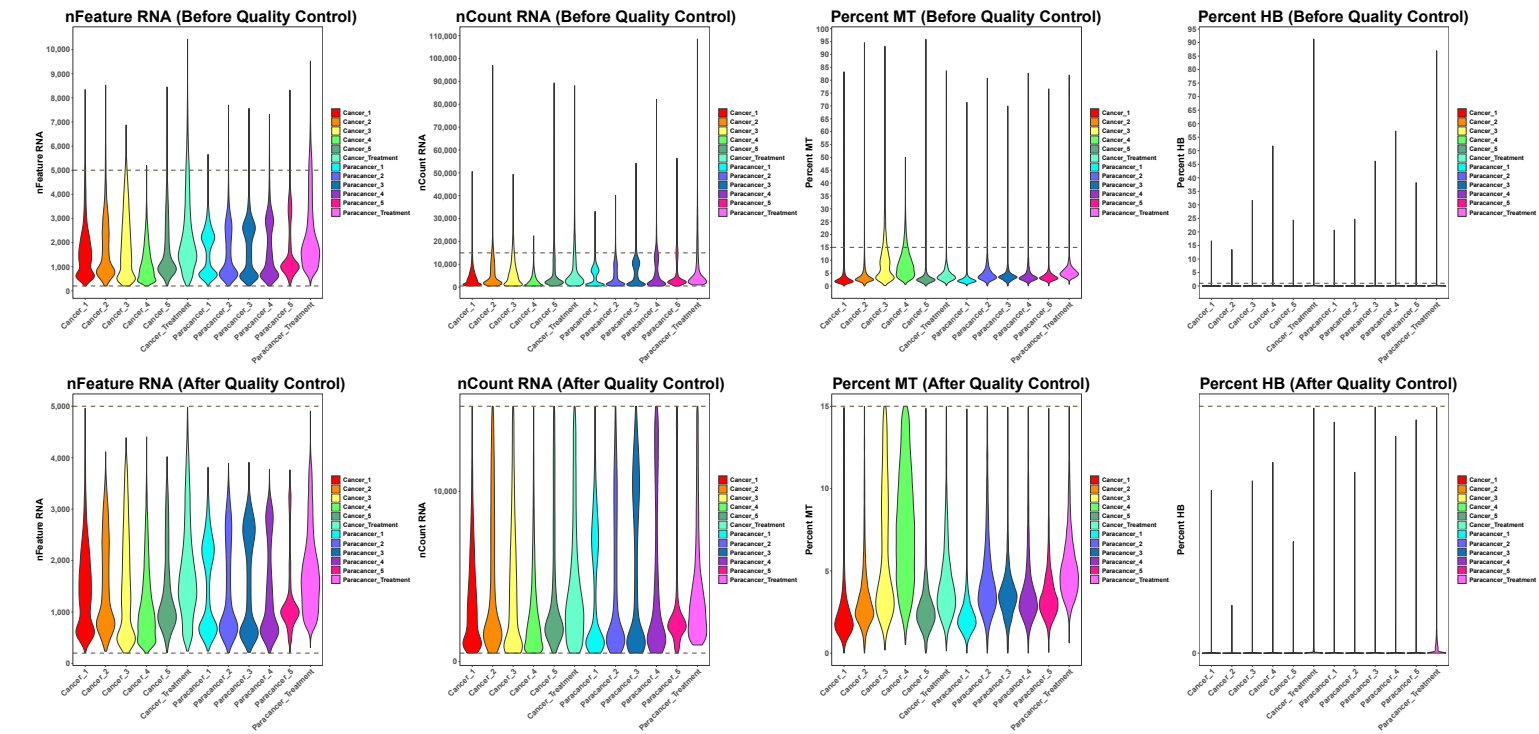**B**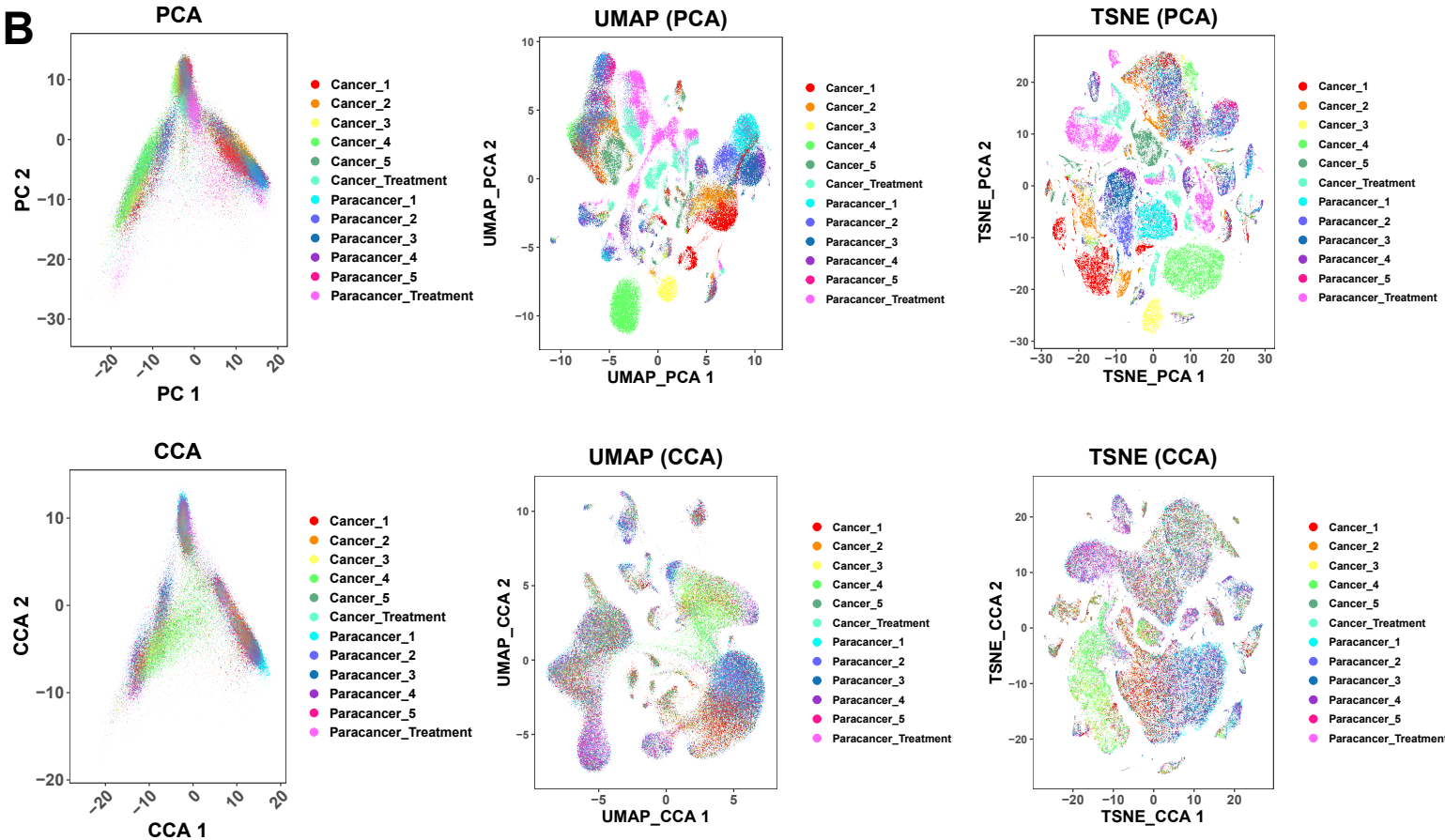**C**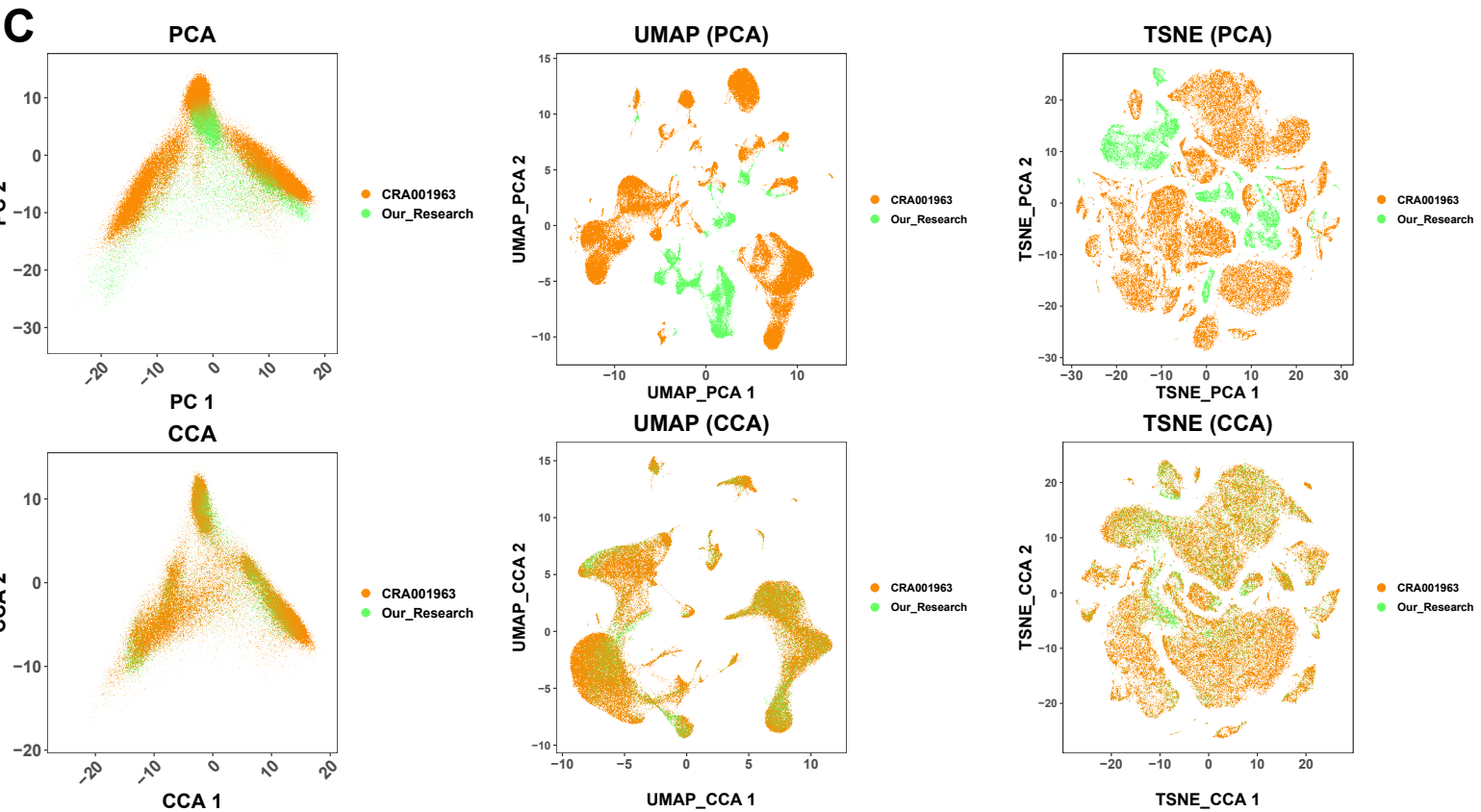

Supplement: Supplementary file 1 — Supplementary file1 (PDF 4348 KB) [file 13258_2025_1706_MOESM1_ESM.pdf]

# Differences in EGFR expression by cell type between cancer and treatment groups

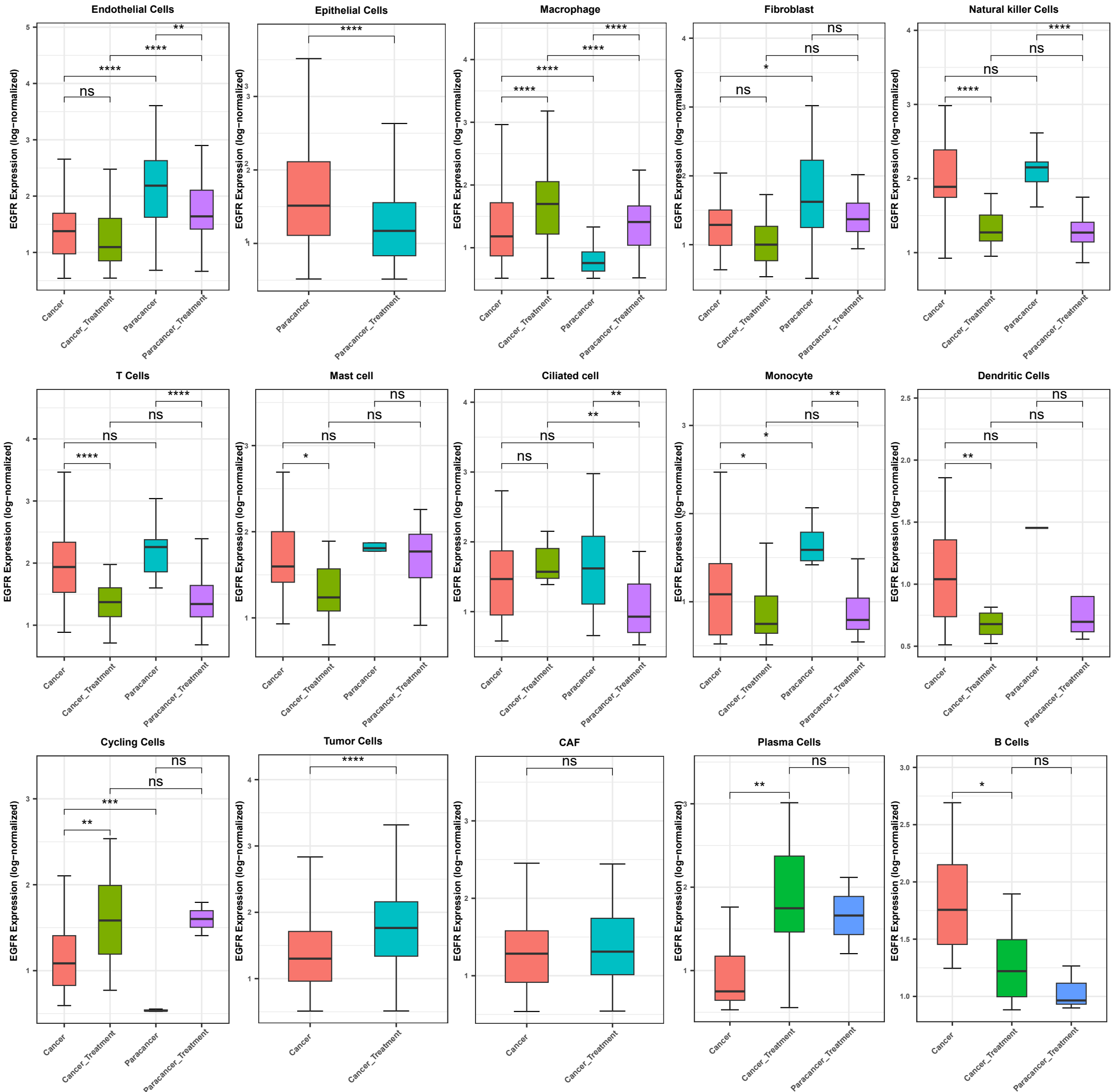

Supplement: Supplementary file 2 — Supplementary file2 (PDF 535 KB) [file 13258_2025_1706_MOESM2_ESM.pdf]

A

FIGAROL\_EGFR\_TKI\_DRUG\_TOLERANT\_CELL\_UP

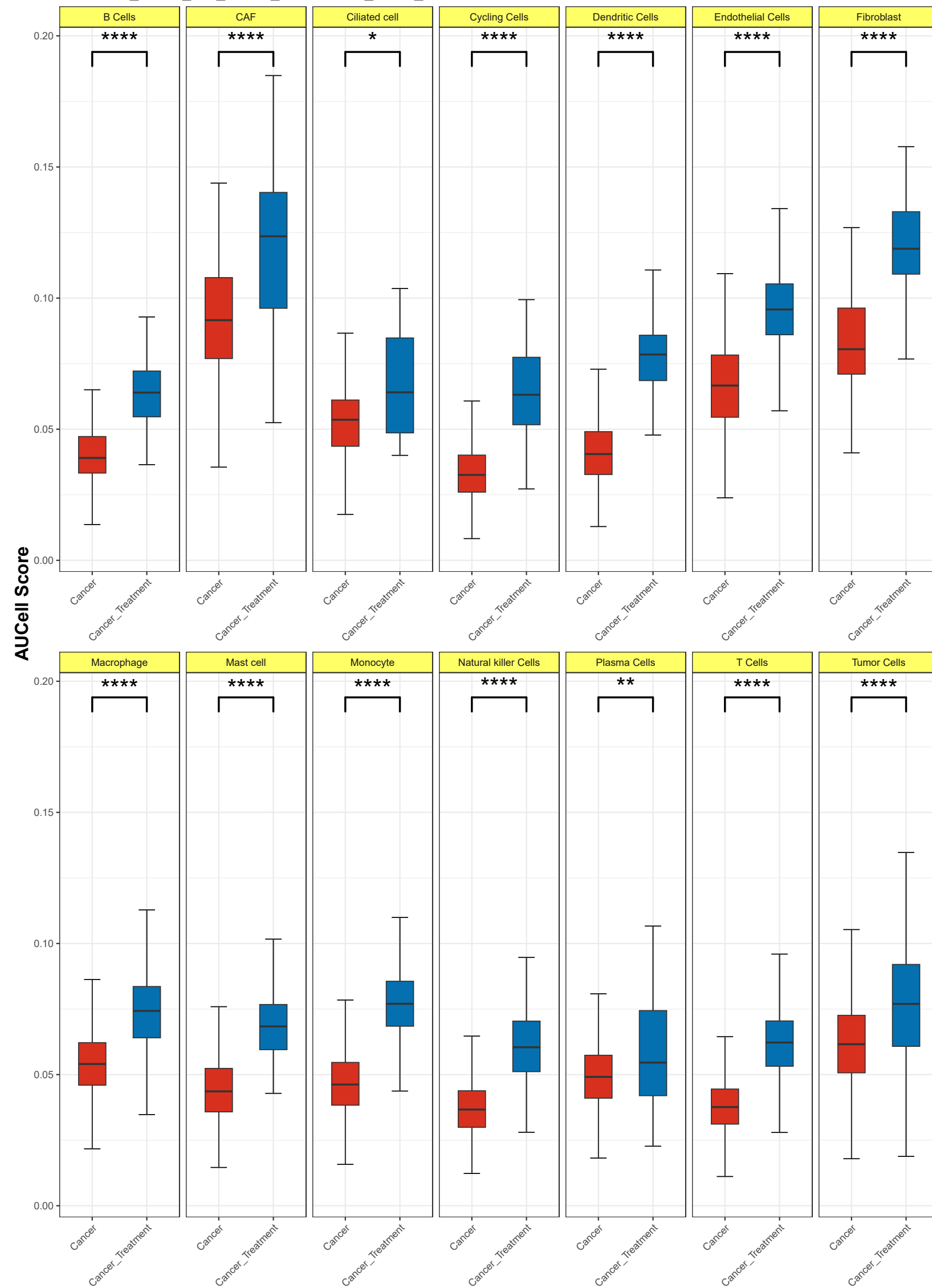

B

FIGAROL\_EGFR\_TKI\_DRUG\_TOLERANT\_CELL\_DN

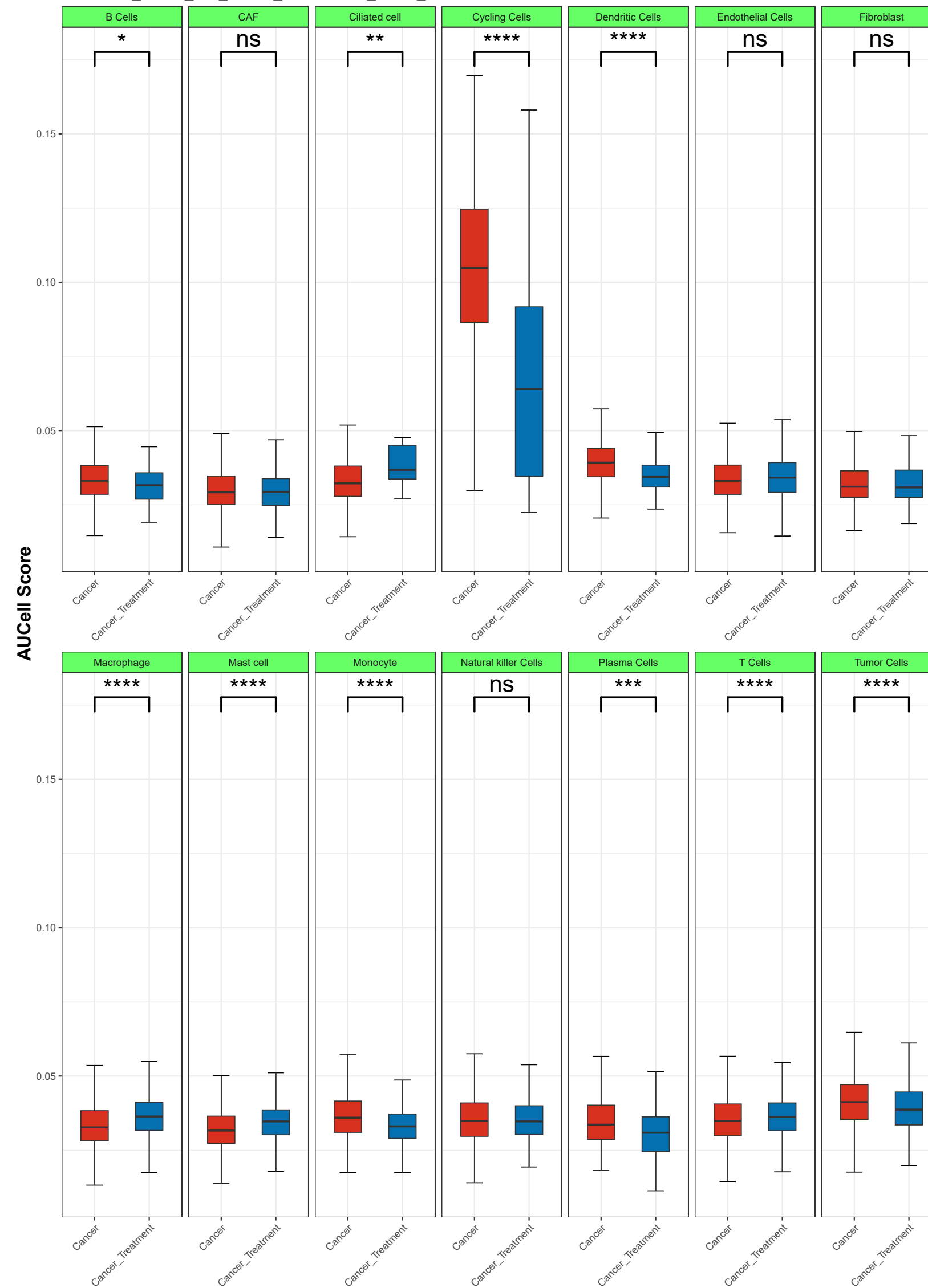

Supplement: Supplementary file 3 — Supplementary file3 (PDF 206 KB) [file 13258_2025_1706_MOESM3_ESM.pdf]
